# The British and Foreign Medico-Chirurgical Review

**Published:** 1860-05

**Authors:** 


*The British and Foreign Medico-Chirurgical Review,* for January, is before us, through the politeness of the American publishers, S. S. & Wm. Wood.

This quarterly journal of British and Foreign Medical literature is indispensable to the buyer of foreign books. Its reviews are distinguished for ability and candor, and the reader is made acquainted with the views of all the more distinguished foreign authors. Its original department always contains able papers, and its *reports* upon the various branches of medical science are generally well made up. O. C. G.

